# Pharmacological Classification and Activity Evaluation of Furan and Thiophene Amide Derivatives Applying Semi-Empirical *ab initio* Molecular Modeling Methods

**DOI:** 10.3390/ijms13066665

**Published:** 2012-05-30

**Authors:** Leszek Bober, Piotr Kawczak, Tomasz Baczek

**Affiliations:** 1 POLPHARMA SA Pharmaceutical Works, Starogard Gdanski 83-200, Poland; E-Mail: leszek.bober@plusnet.pl; 2 Department of Pharmaceutical Chemistry, Faculty of Pharmacy, Medical University of Gdansk, Gdansk 80-416, Poland; E-Mail: p99p@gumed.edu.pl; 3 Department of Health Sciences, Division of Human Anatomy and Physiology, Pomeranian University of Slupsk, Slupsk 76-200, Poland

**Keywords:** furan derivatives, thiophene derivatives, HPLC, molecular modeling, structural analysis

## Abstract

Pharmacological and physicochemical classification of the furan and thiophene amide derivatives by multiple regression analysis and partial least square (PLS) based on semi-empirical *ab initio* molecular modeling studies and high-performance liquid chromatography (HPLC) retention data is proposed. Structural parameters obtained from the PCM (Polarizable Continuum Model) method and the literature values of biological activity (antiproliferative for the A431 cells) expressed as LD_50_ of the examined furan and thiophene derivatives was used to search for relationships. It was tested how variable molecular modeling conditions considered together, with or without HPLC retention data, allow evaluation of the structural recognition of furan and thiophene derivatives with respect to their pharmacological properties.

## 1. Introduction

The process of searching for anticancer drugs involves consideration of different structures with different mechanisms of action. The methods of searching for the optimal structures (drug design, combinatorial synthesis, screening, *etc*.) also vary [1–3].

One can find an interesting approach in the work by Hollósy *et al.* [4] where two series of amide derivatives of 2-furanocarboxylic and 2-thiophenecarboxylic acids were selected for synthesis and testing. The authors obtained two sets of six derivatives of the acids (six pairs of compounds, a derivative of furan and an analogous derivative of thiophene in each pair). In the next step, they established the retention factor, log *k*, as a measure of lipophilicity in isocratic conditions for the studied compounds along with the use of the classical Hansch method [5]. They calculated also the logarithms of the partition coefficients, clog *P*, as an independent measure of lipophilicity. Finally, the authors studied the biological activity, the antiproliferative effect of the A431 cells expressed as LD_50_, and conducted a preliminary assessment of the statistical relationship between biological activity and lipophilicity arriving at a higher consistency for derivatives of thiophene and the calculated lipophilicity parameters (clog *P*).

Pondering the results obtained by the authors [4] we arrived at the question on how lipophilicity of the compounds and their antiproliferative activity related to structural parameters derived under the quantum chemistry calculation methods for molecules, when both isolated (*in vacuo*), and placed in an aqueous medium. If a dependence were to be statistically proven, it could allow an attempt at a preliminary explanation of the mechanism underlying the antiproliferative action of the analyzed compounds.

The object of this study was to conduct a statistical analysis of the data established by computational *ab initio* methods (for both isolated molecules, and molecules in aquatic environment conditions) and the parameters characterising lipophilicity and biological activity as presented in the work by Hollósy *et al*. [4] in order to confirm their chemometric dependence. The aim of this study was to demonstrate the common and the differentiating features of the considered compounds in terms of their physicochemical and pharmacological effects.

## 2. Experimental Procedure

### 2.1. Analytes

**Figure 1:**
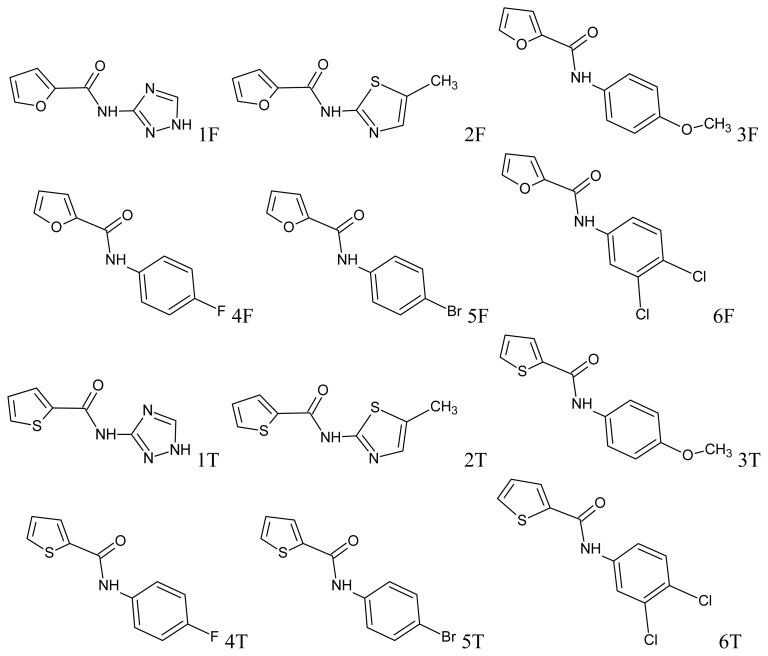
Structural formulas of compounds studied—amide derivatives of furan (1–6 F) and thiophene (1–6 T).

The following compounds were used in the study: *N*-(3-triazolo)-amide furan-2-carboxylic acid (F1), *N*-(5-methyl)-2-amide furan-2-carboxylic acid (F2), *N*-(4-methoxyphenyl)-amide furan-2-carboxylic acid (F3), *N*-(4-fluorophenyl)-amide furan-2-carboxylic acid (F4), *N*-(4-bromophenyl)-amide furan-2-carboxylic acid (F5), *N*-(3,4-dichlorophenyl)-amide furan-2-carboxylic acid (F6), *N*-(3-triazolo)-amide thiophene-2-carboxylic acid (T1), *N*-(5-methylthiazol)-2-amide thiophene-2-carboxylic acid (T2), *N*-(4-methoxyphenyl)-amide thiophene-2-carboxylic acid (T3), *N*-(4-fluorophenyl)-amide thiophene-2-carboxylic acid (T4), *N*-(4-bromophenyl)-amide thiophene-2-carboxylic acid (T5) and *N*-(3,4-dichlorophenyl)-amide thiophene-2-carboxylic acid (T6). All structural formulas of the compounds studied are presented on Figure 1.

### 2.2. Biological Activity Data

The study used the literature-quoted data of biological activity (antiproliferative for A431 cells) expressed as LD_50_. Based on previous experience [6,7] the values presented by the authors [4] were converted to their logarithmic form, log (1/LD_50_). The value of biological activity presented in this form is directly proportional to the force of action, hence better correlated with the structural parameters.

### 2.3. Chromatographic Lipophilicity Data

The chromatographic data were expressed as log *k* and derived from the cited paper [4]. The authors obtained them using the Hypersil MOS (C_8_) column and a mobile phase containing 0.25 M triethylamine phosphate, pH 2.25, and 24% v/v acetonitrile. The analysis further included the semi-empirical values of log *P* (clog *P*) presented by the authors [4] and calculated by the Hansch method [5].

### 2.4. Molecular Descriptors

The non-empirical structural indicators, *i.e*., quantum-chemical indicators, were calculated in the study. The structure of the tested compounds was examined by molecular modeling with the Gaussian 03W software (v03, Gaussian Inc., Wallingford, CT, USA, 2003). The geometry of molecules was optimized using the restricted Hartree-Fock 6–31G (d, p) method [8] also known as 6–31G** and then extended in addition with the method of polarization functions 6–31G (3d, 3p) [8], which assumes that there are three polarization functions—d and three polarization functions—p in the atom. The structure was optimized directly (*in vacuo*) and in the aquatic environment using the PCM method (Polarizable Continuum Model) [9–11].

The quantum-chemical indices considered were as follows: total energy (TE); electronic spatial extent (ESE), which is defined as the area covering the volume around the molecule beyond which electron density is less than 0.001 electron Bohr^−3^ and describes the sensitivity of the molecule to the electric field; the energy of the highest occupied molecular orbital (E_HOMO); the energy of the lowest vacant molecular orbital (E_LUMO), and the energy difference of HOMO and LUMO defined as the energy gap (EG). Moreover, the following values were used: the largest positive charge on the electron atoms (MAX_POS), the largest negative charge on the electron atoms (MAX_NEG), the difference between the largest positive and negative charge (DELTA_Q), the total dipole moment (TDM), and the isotropic polarizability (IPOL).

The values of total energy were expressed in atomic energy units a.u. or Hartree, energies of HOMO, LUMO and the energy gaps were expressed in eV and isotropic polarizability in Bohr^−3^. The values of the electron density and electron charges on atoms were expressed in units of elementary charge (ē), the dipole moment in Debye (D), and the electron spatial extent in eBohr^−3^.

For the optimized structures in aqueous environments the following parameters were also used: the polarized solute-solvent interaction energy (PSSIE), the cavitation energy (CE), the dispersion energy (DE), the repulsion energy (RE), and the total energy of non-electrostatic interaction (Tne), all values in kcal/mol.

### 2.5. Statistical Analysis

The retention data of the compounds studied were related to their structural indicators under stepwise, progressive, and multiparametric regression analysis (multiple regression), and the analysis of partial least squares (PLS) was performed in Statistica 10 (v10, StatSoft, Tulsa, OK, USA, 2011) installed on a personal computer.

## 3. Results and Discussion

The numerical values of all 10 structural parameters derived from the quantum-chemical calculations *in vacuo* with the use of the 6–31G (d, p) method for all 12 examined compounds are presented in Table 1. In Table 2 they are presented with the 6–31G (3d, 3p) method used.

The values of 15 structural parameters derived from the PCM calculations in the aquatic environment using 6–31G (d, p) are presented in Table 3. Table 4 presents the same for the 6–31G (3d, 3p) method.

The values of the parameters determining the lipophilicity and biological activity of the compounds in question are presented in Table 5.

A preliminary comparison, of the structural parameters calculated using the 6–31G (d, p) and 6–31G (3d, 3p) methods reveals that some differences in the values calculated for isolated molecules (*in vacuuo*) and molecules in an aqueous medium (PCM model) are only observed for the electron charge on the atoms (the largest positive charge MAX_POS, the largest negative charge MAX_NEG, and the difference between the charges ΔQ) standing at about 30%, and for IPOL (isotropic polarisability) ranging between 15–20%. Minor differences (within 5%) occur for the values of the total dipole moment. This comparison was only an approximate estimation of the values obtained directly from Gaussian software. In order to evaluate the usefulness of the calculations using 6–31G (d, p) and 6–31G (3d, 3p) it was necessary to conduct a multiregression analysis.

Before initiating the multiregression analysis, the data set was subject to cross-validation in the aggregate PLS analysis and in the traditional manner. *i.e*., by sequential removal of one case followed by a statistical analysis of the remaining 11 cases. Then, the mean values were calculated: the directional factors of the independent variables, the intercept and the regression coefficient based on those cases of the regression dependence where, the independent variables were most likely to repeat. Finally, the obtained values were compared with the values derived from the full set of cases (*n* = 12). The results of the multiregression analysis are presented in Tables 6–12.

Geometry optimization and calculation of the structural parameters of the enlarged function base (3d, 3p instead of d, p) does not yield any significant differences in the resulting multiregression relationships. This may be due to the fact that the structural parameters involving the highest observed differences (MAX_POS, MAX_NEG, ΔQ, and IPOL) do not occur at all, or appear sporadically as a third variable. The values of the structural parameters that have the greatest impact on the empirical parameters generally differ by less than 5%, and in some cases by even less than 1% for both functional bases.

**Figure 2:**
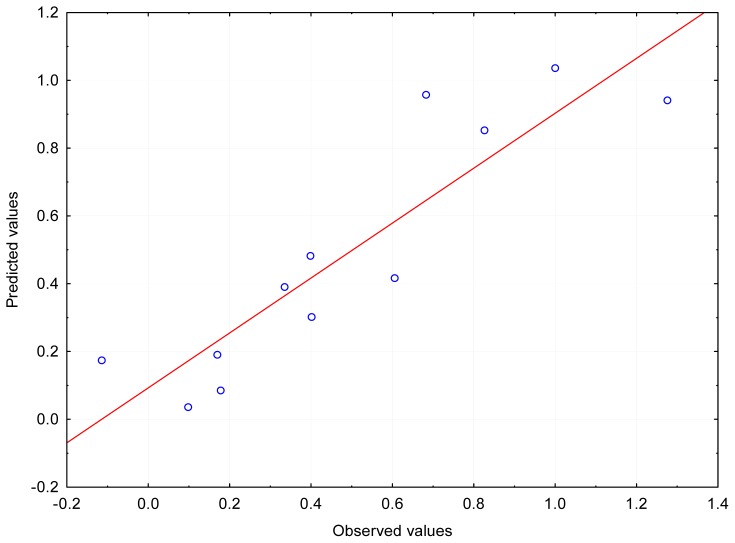
The relationships between observed and predicted values of the structures optimized *in vacuo* using 6–31G (3d, 3p) method; the regression equation: *log k* = *k_0_* *+ k_1_ESE + k_2_TDM*, where *n* = 12, R = 0.9001.

The logarithm of the retention factor, log *k*, for isolated molecules (*in vacuo*) depends primarily on the electron spatial extent (ESE), than on the value of the total dipole moment (TDM). The best agreement (R~0.9) was obtained for those two independent variables (Figure 2). The situation is analogous in aqueous medium (R~0.9). During the cross-validation, dependence was found occasionally for three independent variables: ESE, TDM and MAX_POS (R~0.98) in 11 cases. The logarithm of the partition coefficient, clog *P*, calculated by the authors [4] under the classical Hansch method [5] also shows a very similar dependence for both the isolated molecules and those in the aquatic environment–ESE as a single variable, and ESE with TDM in the case of two variables (R~0.89). The relationship of two variables can be considered satisfactory. Occasionally, for all 12 cases, relationships of three variables were observed: ESE, TDM, and MAX_POS for isolated molecules, and ESE, TDM, and ΔQ for the particles in an aqueous medium (R~0.93–0.94).

The LD_50_ parameter was given for biological activity [4]. This was supplemented with the log (1/LD_50_) parameter being directly proportional to the force of action. The logarithm of the inverse of LD_50_ is proven in having slightly better correspondence with the structural parameters. Isolated molecules (*in vacuo*) demonstrated dependence on only one parameter—the lowest energy unoccupied molecular orbitals (E_LUMO) with R~0.89–0.90 (LD_50_) and R~0.91 (log (1/LD_50_)).

**Figure 3:**
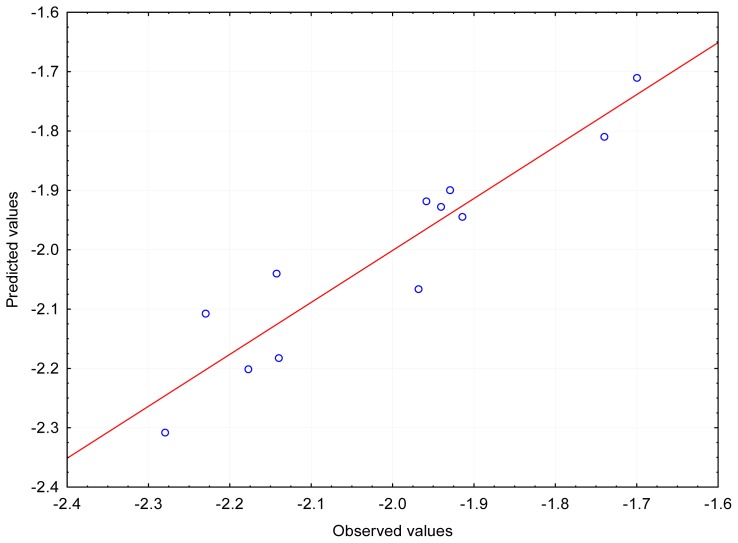
The relationships between observed and predicted values for the structures optimized in the aquatic environment using 6–31G (d, p) method; the regression equation: *log (1/LD_50_) = k_0_* *+ k_1_DE + k_2_E_LUMO*, *n* = 12, R = 0.9355.

When the particles were optimized in the aquatic environment a shift of the structural parameters was recorded. This may be due to the fact that more parameters, not noted for isolated molecules, were taken into account for the aquatic environment. Moreover, there were more variables than cases so the calculation matrix became “oversquare”. The first important variable was the energy of dispersion (ED), although the dependence on E_LUMO for the log (1/LD_50_) occurred sporadically during cross-validation; the, R values were: ~0.83 (LD_50_) and ~0.84 (log (1/LD_50_)). A dependence was also found for two other variables: DE and E_LUMO with R~0.91–0.92 (LD_50_) and R~0.93–0.94 (log (1/LD_50_), presented on Figure 3). In addition, for log (1/LD_50_) a relationship was found between the following three variables: DE, E_LUMO, and the energy of interaction of the polarized solute-solvent (PSSIE), with R~0.96–0.97.

Among the parameters determining the lipophilicity of the molecules (chromatographic, log *k*, and calculated, clog *P*) those of the greatest impact are as follows: electron spatial extent (ESE) reflecting the particles’ dispersion ability and the London force interactions. Coming second in significance is the total dipole moment (TDM) reflecting the targeted electrostatic interactions. Incidentally, the influence of the electrical charges on the atoms (the largest positive difference MAX_POS and maximum positive and negative ΔQ) also appeared to be associated with more local electrostatic interactions. The coefficients of proportionality occurring for the ESE and TDM values are positive, which indicates that lipophilicity is directly proportional to these two parameters. The charges on atoms (or their difference) occasionally appearing in the equations as the third parameter show negative values for the proportionality coefficients, which leads to reduction of lipophilicity.

In the case of the parameters determining antiproliferative activity we observe competition between the energy of lowest unoccupied molecular orbitals and energy of dispersion. The energy of the LUMO orbitals, according to Koopman’s theorem, carries the physical sense of electron affinity (EA). A positive value of the LUMO energy in the thermodynamic convention denotes the energy, which must be supplied to the system in order to attach an additional electron to the molecule or otherwise convert it to an anion). On the other hand, negative LUMO energy denotes the energy provided by the system, which means that the process is exergonic. EA = −E_LUMO, and is the measure of electrophilicity of a molecule that is particularly important in the modeling of molecular properties and reactivity (radical reactions). The energy of dispersion (DE) denotes the share of the dispersive effect energy of the total energy of the solute-solvent interactions. The dispersion energy term is often collated with the repulsion into a unique term defining the so-called van der Waals contribution to the interaction energy of molecules [10,12]. The proportionality coefficients of the two independent variables (E_LUMO and DE) have negative signs (in the dependency for log (1/LD_50_)), the E_LUMO values are positive, while the DE values are negative. The exact numerical values are determined by the values of free expression. Increase of the LUMO energy leads to a reduction in antiproliferative activity, whereas increase of DE leads to growth in the antiproliferative activity. The third and incidentally occurring variable, *i.e*., the energy of the solute-solvent impacts, increases antiproliferative activity just like the energy of dispersion. The interaction energies of the solute and solvent, and particularly the dispersion component in the polar solvent, *i.e*., water, can serve as a model for interactions of substances demonstrating antiproliferative activity of cellular receptors. They represent non-specific interactions. This is indicated in the conclusions of the work [4] about the relationship between lipophilic and biological activities. The meaning of LUMO orbital energy should be interpreted differently. All examined compounds have positive values of LUMO orbital energy, which means that E_LUMO reduces the value of log (1/LD_50_) (the positive value of E_LUMO signifies that no anions are formed). If the E_LUMO values were negative, then the element described by this parameter would be positive and increase the value of log (1/LD_50_) (anions of the molecules in question would form, as well as possible additional interactions with the polar groups and the positively charged cellular structures).

As was shown in the cited work by Hollósy *et al*. [4], for the regressions between the parameters of lipophilicity and biological activity for each separate subset of the compounds under consideration, *i.e*, derivatives of furan and thiophene, it was decided to examine how the presented empirical parameters depend on the structural parameters in each subset. The presented results of those statistical analyses were only indicative and approximate due to the fact that they formed a small subset (*n* = 6) and the compounds contained in them differ in structure to a lesser degree than the full set of compounds.

In the subgroup of the furan derivatives, the lipophilicity parameters for the isolated molecules (*in vacuo*), just like the full set, depend mainly on the electron spatial extent (ESE) with R~0.85–0.90. The antiproliferative activity (only LD_50_ value) depends on the isotropic polarisability (IPOL), with R~0.82–0.85. Thus, the most important independent variables are: the structural parameters determining the possibility of dispersion and London force interactions.

In the aqueous medium all parameters, *i.e*., lipophilicity and biological activity, depend on the dispersive energy (ED), with R~0.93, and R~0.89 for the activity expressed as log (1/LD_50_). The correlation between LD_50_ and ED is therefore slightly closer than the correlation found by the authors [4] between LD_50_ and log *k*.

In the subgroup of the thiophene derivatives the lipophilicity parameters and biological activity for the isolated molecules (*in vacuo*) depend on the energy of the lowest unoccupied molecular orbitals (E_LUMO). However, with LD_50_, dependence on the electron spatial extent (ESE) occurred for the particles optimized on the (d, p) base alongside the two parametric dependencies on the ESE and the energy of the highest unoccupied molecular orbitals (E_HOMO). The regression coefficients characteristic for most of the dependencies are: R~0.85–0.89, R~0.92–0.93 for the log *k* dependencies, and R~0.99 for the two parametric dependencies for LD_50_.

In the aqueous medium all parameters, *i.e*., of both lipophilicity and proliferative activity, depend mainly on the energy of repulsion (repulsion between the solute and solvent particles)—RE. The values of regression coefficients were identified at R~0.93–0.96. The correlation between the biological parameters and the RE was also slightly better than that between the LD_50_ and log *k* presented in the work [4]. Furthermore, two-parametric dependencies developed with isotropic polarisability (IPOL) or total energy of non-electrostatic interactions (Tne) with the values of R~0.98–0.99 coming in as the prevailing second parameter.

The statistical analysis of the compound subsets indicates that the parameters of lipophilicity and biological activity are generally best correlated to the structural parameters describing the effect commonly referred as non-polar interactions, which confirms the conclusions drawn in the work by Hollósy *et al*. [4].

## 4. Concluding Remarks

Based on the above overview of the results the following conclusions can be drawn.

Out of the considered 10 quantum-chemical parameters calculated for the isolated molecules the electron spatial extent (ESE) and the total dipole moment (TDM) prove to have the greatest impact on the lipophilicity parameters, whereas the energy of lowest unoccupied molecular orbitals (E_LUMO) proves to be most determinant of the biological activity.

In the aqueous medium of the PCM model and the considered 15 quantum-chemical parameters, ESE and TDM again proved to have the highest influence on lipophilicity (a third independent variable appearing occasionally). The biological activity, on the other hand, proved to be the closest related to the energy of dispersion (ED) with the E_LUMO variable coming second.

Concerning the subsets, including the furan derivatives and thiophene derivatives: the electron spatial extent (ESE) has the greatest impact on the lipophilicity parameters in the group of isolated furan derivative molecules; whereas isotropic polarisability (IPOL) proves to be the most potent determinant of biological activity. In the aquatic environment the dispersion energy (DE) proved to have the highest impact on both lipophilicity and biological activity.

In the group of thiophene derivatives E_LUMO and repulsion energy (RE) generally appear to have the greatest impact on the parameters of both lipophilicity and biological activity in isolated molecules.

The dependencies on the parameters derived from the quantum-chemical calculations confirm the earlier obtained dependencies between the parameters of lipophilicity and biological activity for the studied compounds.

Most of the quantum-chemical structural parameters, for which the relationship with the empirical parameters was determined, are related to dispersion and London force interactions, sometimes called non-polar interactions.

The structural parameters of the polar character are as follows: the total dipole moment (TDM), which is the second parameter of impact on the parameters of lipophilicity, and the energy of the lowest unoccupied molecular orbitals (E_LUMO) which affects the antiproliferative activity and most likely has an impact on the cellular redox processes.

## Supplementary Materials



## Figures and Tables

**Table 1:** The numerical values of 10 structural parameters derived from quantum-chemical calculations 6–31G (d, p) method *in vacuo* for all 12 analyzed compounds.

Compound	TE	ESE	E_HOMO	E_LUMO	EG	MAX_POS	MAX_NEG	ΔQ	TDM	IsoPol
**1F**	−636.07	2474.06	−8.8656	3.0531	11.9187	0.8235	−0.8791	1.7026	5.7507	87.93
**2F**	−1001.59	3710.12	−8.5739	2.7080	11.2819	0.8006	−0.8270	1.6276	3.1299	115.85
**3F**	−739.85	5591.96	−7.7325	2.9755	10.7080	0.8003	−0.8511	1.6513	3.1471	128.70
**4F**	−724.82	4490.71	−8.3135	2.7878	11.1013	0.8014	−0.8523	1.6537	5.3470	112.43
**5F**	−3195.28	7208.03	−8.3334	2.6120	10.9454	0.8022	−0.8539	1.6562	5.7436	132.32
**6F**	−1543.76	6490.64	−8.6180	2.4552	11.0732	0.8028	−0.8557	1.6584	7.4356	135.08
**1T**	−958.73	2777.61	−8.9954	2.6150	11.6104	0.8444	−0.8786	1.7229	5.5502	100.73
**2T**	−1324.25	4071.48	−8.6898	2.4139	11.1037	0.8321	−0.8227	1.6548	3.1543	129.02
**3T**	−1062.52	6319.25	−7.7668	2.4014	10.1682	0.8648	−0.8471	1.7119	3.0444	141.97
**4T**	−1047.49	5109.75	−8.3560	2.2433	10.5993	0.8660	−0.8484	1.7144	5.1907	125.55
**5T**	−3517.94	8139.24	−8.3799	2.1845	10.5644	0.8504	−0.8388	1.6892	5.5362	145.53
**6T**	−1866.43	7328.30	−8.6561	2.0044	10.6605	0.8674	−0.8521	1.7195	7.2734	148.25

**Table 2:** The numerical values of 10 structural parameters derived from quantum-chemical calculations 6–31G (3d, 3p) method *in vacuo* for all 12 analyzed compounds.

Compound	TE	ESE	E_HOMO	E_LUMO	EG	MAX_POS	MAX_NEG	ΔQ	TDM	IsoPol
**1F**	−636.11	2472.36	−8.8618	2.9557	11.8175	1.1067	−1.1117	2.2184	5.6098	100.19
**2F**	−1001.63	3705.85	−8.5320	2.6370	11.4690	1.0726	−1.1213	2.1939	3.1582	130.05
**3F**	−739.90	5583.02	−7.7293	2.9233	10.6526	1.2402	−1.2044	2.4446	3.1555	145.45
**4F**	−724.86	4483.29	−8.3124	2.7255	11.0379	1.2511	−0.9106	2.1617	5.2793	127.78
**5F**	−3195.37	7188.26	−8.3048	2.5410	10.8458	1.2533	−0.9057	2.1591	5.6901	151.90
**6F**	−1543.80	6478.47	−8.5467	2.4014	10.9481	1.2472	−0.9830	2.2303	7.1568	156.68
**1T**	−958.77	2772.40	−8.9709	2.5565	11.5274	1.0668	−0.8035	1.8704	5.4021	114.11
**2T**	−1324.30	4066.11	−8.6341	2.3717	11.0058	0.8975	−0.7826	1.6801	3.1599	144.29
**3T**	−1062.56	6305.01	−7.7687	2.4044	10.1731	1.1166	−1.2039	2.3205	3.0491	159.76
**4T**	−1047.53	5097.75	−8.3606	2.2392	10.5998	1.1282	−0.8523	1.9805	5.1112	141.92
**5T**	−3518.04	8111.85	−8.3584	2.1611	10.5195	1.1441	−0.8771	2.0212	5.4787	165.98
**6T**	−1866.47	7311.38	−8.5883	2.0071	10.5954	1.1264	−0.8363	1.9626	6.9842	170.90

**Table 3:** The numerical values of 15 structural parameters derived from quantum-chemical calculations 6–31G (d, p) method in water for all 12 analyzed compounds.

Compound	TE	ESE	E_HOMO	E_LUMO	EG	MAX_POS	MAX_NEG	ΔQ	TDM	IsoPol	PSSIE	CE	DE	RE	Tne
**1F**	−636.10	2476.66	−8.9881	2.7619	11.7500	0.8229	−0.8806	1.7036	7.6457	113.82	−25.85	23.55	−18.42	1.60	6.73
**2F**	−1001.61	3716.68	−8.5840	2.5067	11.0907	0.8173	−0.8304	1.6477	3.7271	151.21	−16.24	28.29	−20.49	1.51	9.31
**3F**	−739.88	5597.17	−7.8667	2.8430	10.7097	0.8075	−0.8491	1.6566	5.1573	165.53	−21.29	31.07	−21.70	1.45	10.81
**4F**	−724.85	4494.59	−8.2972	2.7739	11.0711	0.8103	−0.8508	1.6611	7.6968	146.84	−20.90	27.38	−20.73	1.61	8.25
**5F**	−3195.30	7215.63	−8.3282	2.6234	10.9516	0.8125	−0.8529	1.6754	8.1613	173.90	−21.10	29.10	−22.84	1.86	8.13
**6F**	−1543.79	6494.17	−8.5334	2.5143	11.0477	0.8153	−0.8551	1.6704	10.5628	176.88	−22.14	30.57	−23.93	2.01	8.65
**1T**	−958.76	2775.58	−9.1709	2.3586	11.5295	0.8521	−0.8797	1.7318	7.3680	134.38	−25.05	24.23	−20.12	1.75	5.86
**2T**	−1324.26	4067.55	−8.6251	2.2294	10.8545	0.8480	−0.8244	1.6724	3.7027	172.15	−15.15	28.91	−22.11	1.65	8.45
**3T**	−1062.53	6326.05	−7.8915	2.3497	10.2412	0.8704	−0.8467	1.7170	5.2606	186.30	−20.00	31.96	−23.50	1.65	10.10
**4T**	−1047.51	5114.34	−8.3391	2.3029	10.6420	0.8734	−0.8485	1.7219	7.7298	167.51	−19.37	28.27	−22.52	1.80	7.55
**5T**	−3517.97	8114.71	−8.4539	2.2653	10.7192	0.8564	−0.8266	1.6830	8.1202	193.59	−20.15	30.04	−24.66	2.09	7.47
**6T**	−1866.45	7332.67	−8.5862	2.1467	10.7329	0.8787	−0.8528	1.7315	10.5812	197.49	−20.78	31.46	−25.72	2.20	7.94

**Table 4:** The numerical values of 15 structural parameters derived from quantum-chemical calculations 6–31G (3d, 3p) method in water for all 12 analyzed compounds.

Compound	TE	ESE	E_HOMO	E_LUMO	EG	MAX_POS	MAX_NEG	ΔQ	TDM	IsoPol	PSSIE	CE	DE	RE	Tne
**1F**	−636.15	2473.06	−8.9785	2.6667	11.6452	1.1010	−1.1354	2.2365	7.7255	134.88	−25.71	23.53	−18.42	1.60	6.71
**2F**	−1001.65	3709.09	−8.5494	2.4242	10.9736	1.0307	−1.1462	2.1769	3.9076	175.92	−16.26	28.26	−20.48	1.50	9.28
**3F**	−739.92	5586.89	−7.8599	2.7799	10.6398	1.2236	−1.2102	2.4338	5.3148	192.94	−21.07	31.04	−21.70	1.45	10.79
**4F**	−724.89	4486.57	−8.2969	2.7045	11.0014	1.2371	−1.0071	2.2442	7.8054	172.28	−21.04	27.35	−20.73	1.60	8.23
**5F**	−3195.40	7192.27	−8.2934	2.5472	10.8406	1.2440	−1.0070	2.2510	8.2618	207.22	−20.82	29.05	−22.83	1.86	8.08
**6F**	−1543.83	6482.36	−8.4792	2.4395	10.9187	1.2439	−1.0078	2.2517	10.4804	214.32	−21.84	30.54	−23.92	2.00	8.62
**1T**	−958.81	2768.47	−9.1388	2.3061	11.4449	1.0647	−0.8626	1.9273	7.4300	158.67	−24.87	24.20	−20.12	1.75	5.83
**2T**	−1324.32	4059.54	−8.5837	2.1815	10.7652	0.8650	−0.8381	1.7032	3.8445	199.93	−15.17	28.87	−22.11	1.65	8.41
**3T**	−1062.59	6311.51	−7.8866	2.3355	10.2221	1.1045	−1.2101	2.3146	5.3958	216.82	−19.71	31.92	−23.50	1.64	10.06
**4T**	−1047.55	5101.58	−8.3426	2.2822	10.6248	1.1179	−0.9097	2.0276	7.8160	196.09	−19.61	28.23	−22.51	1.80	7.51
**5T**	−3518.06	8047.83	−8.5146	2.2754	10.7900	1.1365	−0.9033	2.0398	8.2146	228.33	−20.31	30.02	−24.65	2.10	7.47
**6T**	−1866.49	7317.06	−8.5298	2.1233	10.6531	1.1253	−0.8986	2.0238	10.4847	238.03	−20.47	31.42	−25.72	2.20	7.90

**Table 5:** The values of parameters of lipophilicity: the experimental one (log *k*). and calculated (clog *P*), and biological activity, expressed as the LD_50_ and the logarithm of inverse of LD_50_ (log (1/LD50)) value of antiproliferative activity for A431 cells.

Compound	log *k*	clog *P*	LD_50_ (μg/mL)	log (1/LD_50_)
**1F**	−0.115	1.211	190	−2.279
**2F**	0.098	1.547	170	−2.230
**3F**	0.401	2.470	138	−2.140
**4F**	0.607	2.975	150	−2.177
**5F**	0.827	3.477	139	−2.143
**6F**	1.275	4.015	82	−1.914
**1T**	0.169	1.901	93	−1.968
**2T**	0.177	1.741	91	−1.959
**3T**	0.335	2.097	85	−1.929
**4T**	0.398	2.227	87	−1.940
**5T**	0.682	2.947	55	−1.740
**6T**	1.001	3.480	50	−1.699

**Table 6:** The relationships for the structures optimized *in vacuo* and in the aquatic environment; statistical parameters: *R*, *s*, *F* and *P* of regression equation log *k* = *k_0_* *+ k_1_ESE + k_2_TDM*, where *n* = 12, for the series of model compounds.

*k_1_*	*k_2_*	*R*	*s*	*F*	*P*
***in vacuo*** **6–31G (d, p)**					
0.7822 ± 0.1970	-	0.7822	0.2645	15.7659	0.0026
0.6449 ± 0.1508	0.4689 ± 0.1508	0.9016	0.1936	19.5564	0.0005
***in vacuo*** **6–31G (3d, 3p)**					
0.7826 ± 0.1969	-	0.7826	0.2643	15.8042	0.0026
0.6431 ± 0.1522	0.4661 ± 0.1522	0.9001	0.1950	19.2132	0.0006
**aquatic 6–31G (d, p)**					
0.7831 ± 0.1967	-	0.7831	0.2640	15.8536	0.0026
0.5696 ± 0.1645	0.4858 ± 0.1645	0.8964	0.1983	18.4121	0.0006
**aquatic 6–31G (3d, 3p)**					
0.7850 ± 0.1959	-	0.7850	0.2630	16.0537	0.0025
0.5726 ± 0.1652	0.4808 ± 0.1652	0.8957	0.1990	18.2513	0.0007

**Table 7:** The relationships for the structures optimized *in vacuo*; statistical parameters: *R*, *s*, *F* and *P* of regression equation clog *P* = *k_0_* *+ k_1_ESE + k_2_TDM* + *k3MAX_POS*, where *n* = 12, for the series of model compounds.

*k_1_*	*k_1_*	*k_3_*	*R*	*s*	*F*	*P*
***in vacuo*** **6–31G (d, p)**						
0.7778 ± 0.1988	-	-	0.7778	0.5753	15.3119	0.0029
0.6441 ± 0.1576	0.4566 ± 0.1576	-	0.8919	0.4363	17.5052	0.0008
0.7053 ± 0.1250	0.4692 ± 0.1229	−0.3139 ± 0.1201	0.9432	0.3398	21.5098	0.0003
***in vacuo*** **6-31G (3d, 3p)**						
0.7781 ± 0.1986	-	-	0.7781	0.5750	15.3420	0.0029
0.6412 ± 0.1580	0.4570 ± 0.1580	-	0.8919	0.4362	17.5094	0.0008

**Table 8:** The relationships for the structures optimized in the aquatic environment; statistical parameters: *R*, *s*, *F* and *P* of regression equation clog *P* = *k_0_* *+ k_1_ESE + k_2_TDM* + *k3ΔQ*, where *n* = 12, for the series of model compounds.

*k_1_*	*k_1_*	*k_3_*	*R*	*s*	*F*	*P*
**aquatic 6–31G (d, p)**						
0.7786 ± 0.1984	-	-	0.7786	0.5743	15.3962	0.0028
0.5681 ± 0.1695	0.4790 ± 0.1695	-	0.8896	0.4407	17.0703	0.0009
0.4928 ± 0.1378	0.6419 ± 0.1492	−0.3368 ± 0.1340	0.9399	0.3495	20.1994	0.0004
**aquatic 6–31G (3d, 3p)**						
0.7803 ± 0.1695	-	-	0.7803	0.5724	15.5705	0.0027
0.5697 ± 0.1959	0.4769 ± 0.1695	-	0.8899	0.4400	17.1306	0.0008

**Table 9:** The relationships for the structures optimized *in vacuo*; statistical parameters: *R*, *s*, *F* and *P* of regression equation LD_50_ = *k_0_* *+ k_1_E_LUMO*, where *n* = 12, for the series of model compounds.

*k_1_*	*R*	*s*	*F*	*P*
***in vacuo*** **6–31G (d, p)**				
0.8978 ± 0.1393	0.8978	20.8614	41.5386	0.0001
***in vacuo*** **6–31G (3d, 3p)**				
0.8876 ± 0.1457	0.8876	21.8155	37.1293	0.0001

**Table 10:** The relationships for the structures optimized in the aquatic environment; statistical parameters: *R*, *s*, *F* and *P* of regression equation LD_50_ = *k_0_* *+ k_1_DE + k_2_E_LUMO*, where *n* = 12, for the series of model compounds.

*k_1_*	*k_2_*	*R*	*s*	*F*	*P*
**aquatic 6–31G (d, p)**					
0.8298 ± 0.1765	-	0.8298	26.4313	22.1060	0.0008
0.5274 ± 0.1620	0.5023 ± 0.1620	0.9216	19.3734	25.3801	0.0001
**aquatic 6–31G (3d, 3p)**					
0.8301 ± 0.1763	-	0.8301	26.4053	22.1691	0.0008
0.5722 ± 0.1644	0.4593 ± 0.1644	0.9130	20.3672	22.5352	0.0003

**Table 11:** The relationships for the structures optimized *in vacuo*; statistical parameters: *R*, *s*, *F* and *P* of regression equation log (1/LD_50_) = *k_0_* *+ k_1_E_LUMO*, where *n* = 12, for the series of model compounds.

*k_1_*	*R*	*s*	*F*	*P*
***in vacuo*** **6–31G (d, p)**				
−0.9141 ± 0.1282	0.9141	0.0788	50.8209	0.0001
***in vacuo*** **6–31G (3d, 3p)**				
−0.9068 ± 0.1333	0.9068	0.0819	46.2899	0.0001

**Table 12:** The relationships for the structures optimized in the aquatic environment; statistical parameters: *R*, *s*, *F* and *P* of regression equation log (1/LD_50_) *= k_0_* *+ k_1_DE + k_2_E_LUMO + PSSIE*, where *n* = 12, for the series of model compounds.

*k_1_*	*k_1_*	*k_3_*	*R*	*s*	*F*	*P*
**aquatic 6-31G (d, p)**						
−0.8440 ± 0.1696	-	-	0.8440	0.1043	24.7644	0.0006
−0.5398 ± 0.1475	−0.5053 ± 0.1475	-	0.9355	0.0724	31.5427	0.0001
−0.5674 ± 0.1138	−0.5900 ± 0.1177	−0.2646 ± 0.0984	0.9666	0.0556	38.0013	0.0001
**aquatic 6-31G (3d, 3p)**						
−0.8444 ± 0.1694	-	-	0.8444	0.1041	24.8440	0.0005
−0.5844 ± 0.1509	−0.4629 ± 0.1509	-	0.9272	0.0768	27.5695	0.0001
−0.6239 ± 0.1196	−0.5482 ± 0.1232	−0.2756 ± 0.1074	0.9607	0.0603	31.9639	0.0001
