# Supplementary material for: Pharmacological Classification and Activity Evaluation of Furan and Thiophene Amide Derivatives Applying Semi-Empirical ab initio Molecular Modeling Methods
Source: Int J Mol Sci. 2012 May 30;13(6):6665–78. doi: 10.3390/ijms13066665 (PMC3397488; doi:10.3390/ijms13066665)

Pharmacological Classification and Activity Evaluation of Furan and Thiophene Amide Derivatives Applying Semi-Empirical *ab initio* Molecular Modeling Methods

Supplementary Materials

Classification of the furan and thiophen derivatives based on molecular modeling studies and high-performance liquid chromatography (HPLC) retention data was proposed. Structural parameters and the literature values of biological antiproliferative activity of examined compounds was used to search for relationships. It was tested how the variable molecular modeling conditions with or without HPLC retention data considered together allow to evaluate the structural recognition of furan and thiophene amide derivatives in view of their pharmacological properties.

R

X = O-Furan derivatives

X = S-Thiophene derivatives

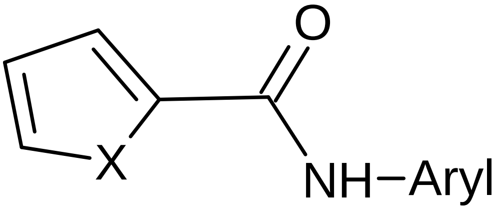

Aryl

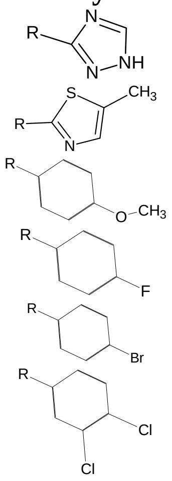

Supplement: Supplementary file 1 [file ijms-13-06665-s001.pdf]
